# Light a lamp and look at the stock market

**DOI:** 10.1186/s40854-021-00232-6

**Published:** 2021-03-22

**Authors:** Radeef Chundakkadan

**Affiliations:** Department of Liberal Arts, Indian Institute of Technology Bhilai, Raipur, Chhattisgarh 492015 India

**Keywords:** Event effect, Investor sentiment, Stock market, Behavioral finance, Lockdown, Covid-19

## Abstract

In this study, we investigate the impact of the light-a-lamp event that occurred in India during the COVID-19 lockdown. This event happened across the country, and millions of people participated in it. We link this event to the stock market through investor sentiment and misattribution bias. We find a 9% hike in the market return on the post-event day. The effect is heterogeneous in terms of beta, downside risk, volatility, and financial distress. We also find an increase (decrease) in long-term bond yields (price), which together suggests that market participants demanded risky assets in the post-event day.

## Introduction

The Coronavirus (COVID-19) outbreak in China at the end of 2019 spread around the globe and infected millions of people. The World Health Organization (WHO) declared COVID-19 a pandemic on March 11, 2020, and several countries opted for nationwide lockdowns. As a result, economic activities were adversely affected, and major stock markets indices plunged (IMF 2020; Zhang et al. 2020; Phan and Narayan 2020). The case of India was not different; the government declared a lockdown and imposed social distancing and isolation measures. As a consequence, on the one hand, economic growth projection fell to 1.9%, and the stock market was drastically affected (IMF 2020; Mishra et al. 2020). On the other hand, the lockdown affected the mental health of people because of (1) the loss of livelihood or lack of employment, and (2) depressing news on COVID-19.^1^

During this challenging time, the government of India called for the light-a-lamp event. This event urged people to switch off all of the lights in their house and to light a lamp, candle, or mobile flashlight for nine minutes. The aim of the event was to express solidarity in the fight against the pandemic, and a vast multitude of the population participated. We hypothesize that the event may have provided psychological relief to the people during the pandemic period and that their positive mood might have been reflected in the stock market. More specifically, we link the event to stock market behavior.

**Fig. 1:**
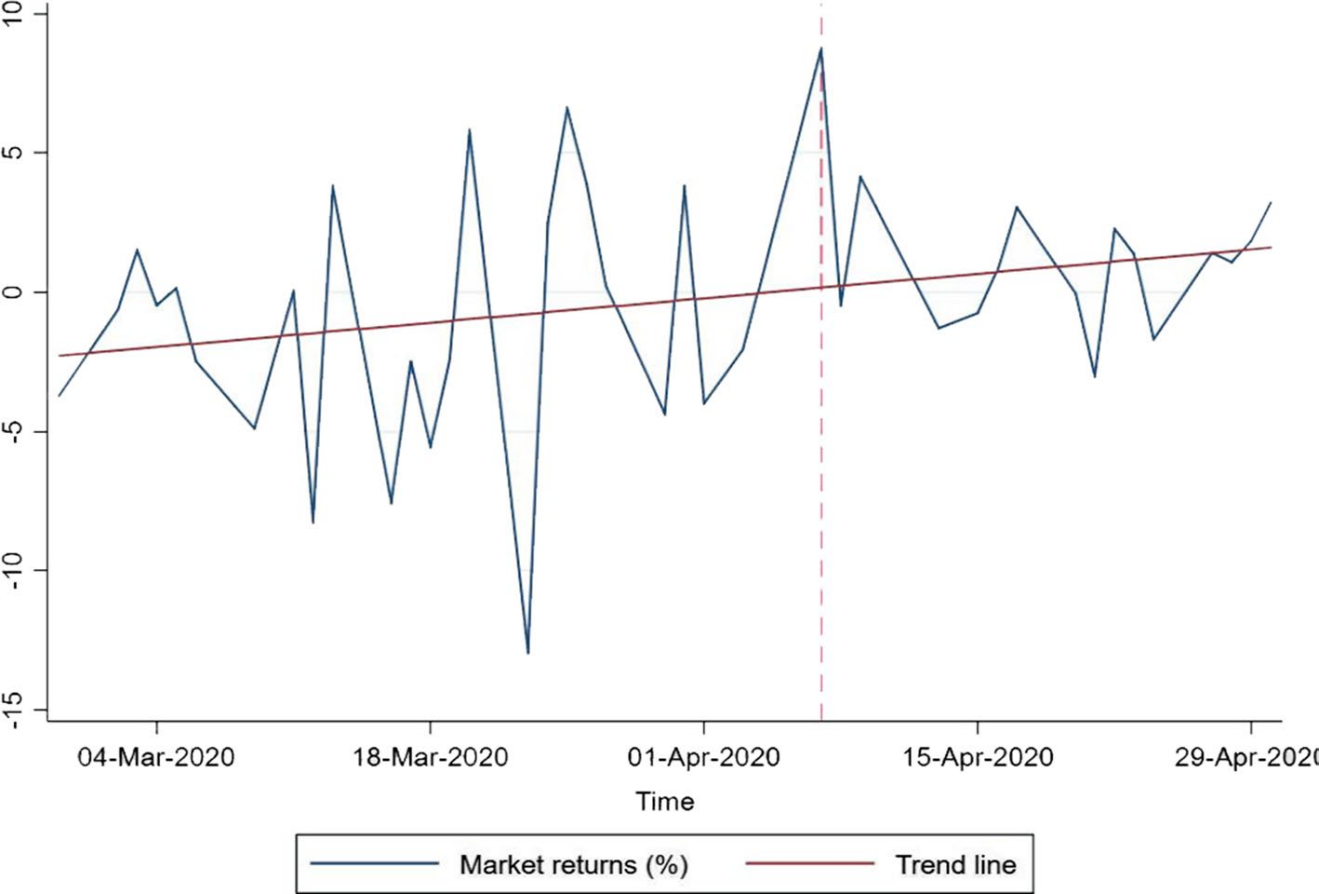
Market returns around the event. *Note*: This figure shows the market returns around *light a lamp* event. The National Stock Exchange (NSE)’s benchmark index, Nifty 50, is used to compute the market returns. The horizontal dashed line indicates the post-event days

The motivation of this study stems from the investor sentiment and the misattribution bias literature. People’s mood can influence their judgments and risk-taking behavior, which in turn can be reflected in their financial decision-making (Johnson and Tversky 1983; Hirshleifer 2001; Baker and Wurgler 2007; DellaVigna 2009). In the stock market literature, existing studies show that events such as sports (Edmans et al. 2007), terrorist activities (Drakos 2010), weather and climate (Saunders 1993; Hirshleifer and Shumway 2003; Kamstra et al. 2003; Chang et al. 2008), and aviation disasters (Kaplanski and Levy 2010; Akyildirim et al. 2020) have an impact on people’s mood and significantly influence the daily stock market returns. In the present case, the light-a-lamp event may have changed people’s mood either by their participation or by watching the media coverage, including on social media. Existing studies provide ample evidence that light and involvement in physical activities play a crucial role in elevating people’s mood (see, Leppämäki et al. 2002; Stephenson et al. 2012; Fernandez et al. 2018). We believe the light-a-lamp event may have reduced people’s stress and anxiety during the lockdown period, which, in turn, may have a positive effect on investment decisions in risky assets. Our study is further motivated as shown in Fig. 1, which presents the market returns around the event day. The horizontal line represents the post-event day. We observe that, between March and April 2020, the stock market experienced the highest spike immediately after the event. The market return on April 7, 2020 (the trading day after the event) is 8.76%, which is much higher than the − 0.55% average returns on other days.

Our first set of analyses investigates whether the light-a-lamp event helps to reduce stress and anxiety among market participants and influences the stock market positively. If the light-a-lamp event generates positive emotions and good mood among the investors, then we expect to observe a positive stock market response in the post-event days. On the contrary, the positive relationship between people’s mood and stock returns cannot be generalized globally (Pizzutilo and Roncone 2017). If the stock market behaves efficiently in accordance with economic fundamentals, then the event has an insignificant effect on the returns (see, Fama 1970; Fama and French 2015). Using daily market returns data for the period from March 1 to April 30, 2020, we present evidence for sentiment-driven stock market movements. Our econometric analysis suggests that there is a 9% hike in the returns on the next trading day of the event.

There is a broad consensus that sentiment-led stock movements reverse in the following days (Tetlock 2007; Garcia 2013; Da et al. 2015). If the market gain is due to the actual economic benefit resulting from the event, then we do not expect to find a reversal. But, if the change in the returns is due to the mood and emotions, we would expect to see an immediate reversal. Since the event under study does not have explicit economic significance, we expect to see a price reversal in the following days. Following Da et al. (2015), we test the price reversal for the following 5 days. Indeed, we find significant evidence for a reversal on the fourth day after the event. One possible explanation for the reversal is that market participants may react immediately after the event and return to their regular trading behavior after a few days (Lee et al. 1991; Kaplanski and Levy 2010). Another explanation, according to cognitive ease and cognitive strain theories (Kahneman 2011), is that investors may have made mistakes in a relaxed environment after the event and then rectified this mistake in the following days.

**Fig. 2:**
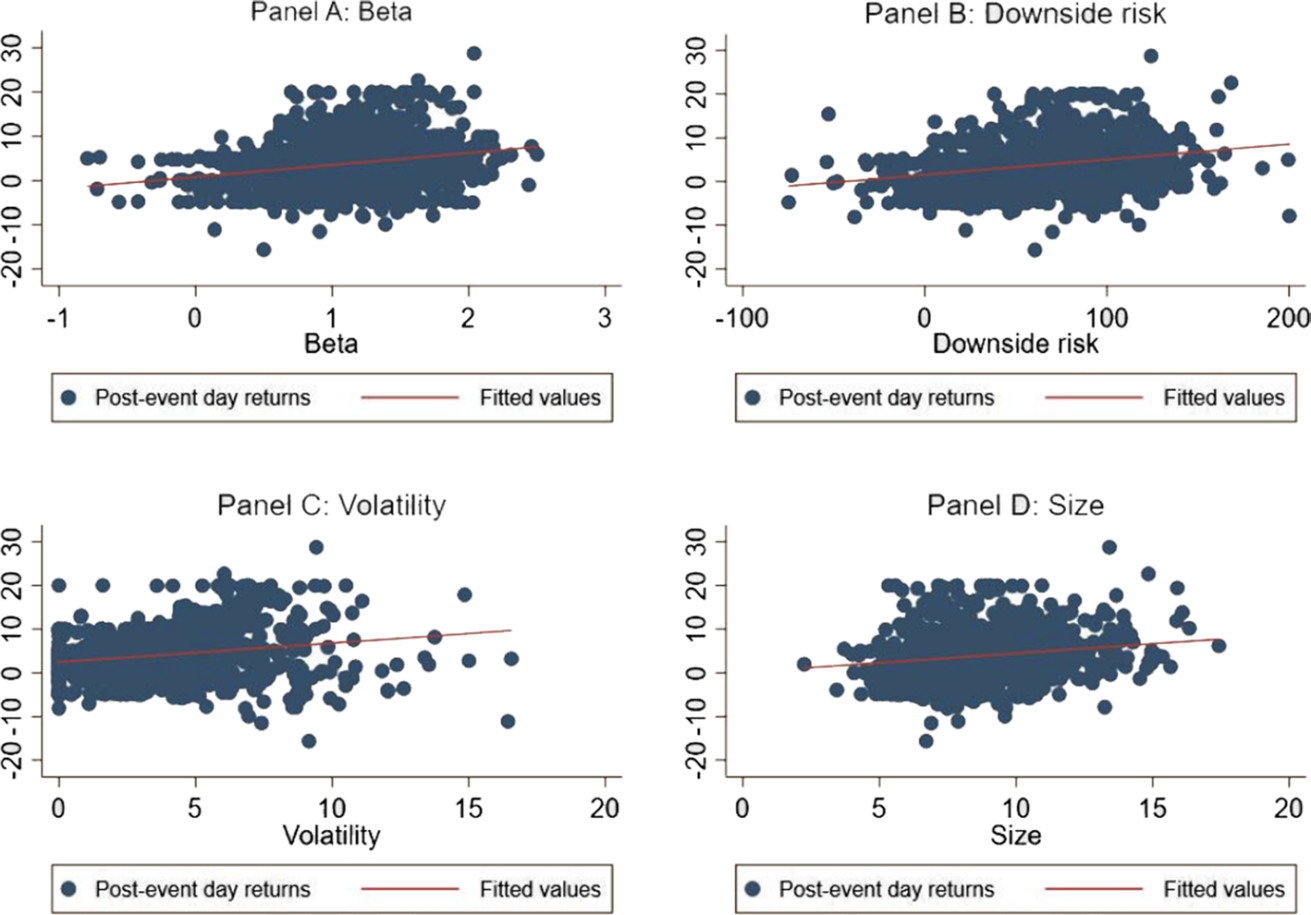
Heterogeneous effect of the event. *Note*: This figure shows the relationship between post-event returns and stock characteristics such as beta, downside risk, volatility and size in Panel A, B, C, and D respectively

Furthermore, the effect of sentiments may not be uniform across the stocks. This effect will be higher for assets with valuations that are highly subjective and difficult to arbitrage (Shleifer and Vishny 1997; Baker and Wurgler 2006, 2007). In that case, we suspect that the magnitude of sentiment effect of the light-a-lamp event will be different for stocks with a higher degree of so-called limit to arbitrage. Figure 2 shows the relationship between post-event returns and stock characteristics. We use beta, downside risk, volatility, and size as stock characteristics in Panel A, B, C, and D, respectively. A clear-cut heterogeneity is evident in the returns. A higher beta, downside risk, volatility, and size are associated with a higher return on the next day of the event. We analyze these relationships more rigorously using econometric tools.

Using firm-level data, we first examine the differential impact of sentiment on stocks with different beta. The stocks with a higher beta are more subject to speculative trading of sentimental investors (Baker et al. 2011). As a result, they may be unattractive to arbitrageurs who take advantage of investors’ irrational behavior. We find that stocks with one standard deviation higher beta are associated with a 1.45% higher return on the immediate day of the event. Since the standard capital asset pricing model (CAPM) beta does not account for the downside risk (Ang et al. 2006), we follow Bawa and Lindenberg (1977) to construct “downside beta” as a proxy for downside risk. Our empirical findings reveal that a one standard deviation increase in the downward risk is associated with 2.4% higher returns on the immediate day of the event. Another heterogeneous effect that we explore is in terms of return volatility. Existing studies show that the sentimental effect is more substantial on a stock with higher volatility (Wurgler and Zhuravskaya 2002; Da et al. 2015). Therefore, we examine the differential impact of light-a-lamp event sentiments on returns of low- and high-volatility stocks. On the next day of the event, there is a 1.45% higher increase in the stocks with one standard deviation higher volatility.

The magnitude of sentimental effects is size dependent. Kaplanski and Levy (2010) find the fact that the effect is more prominent in small firms. We explore this aspect in the context of our event under study. In contrast to existing studies, our empirical estimation suggests that larger firms gained more returns than smaller firms because of the light-a-lamp event. This result may have been due to investors bidding foremost on popular or financially wealthy firms during this time of huge uncertainty. To validate this proposition, we employ several measures of financial distress, such as cashflow, dividend payment, Kaplan and Zingales (1997) index, and Whited and Wu (2006) index, instead of the firm size in the model. As expected, we find that the demand for financially sound firms is higher in the post-event period, irrespective of which distress measure is used.

As a final set of analyses, we examine whether the investors’ response to the event is at the expense of other financial markets. Since the event stimulates a positive mood and emotions among investors, their risk-taking tolerance also will rise (see Bethke et al. 2017). In that case, market participants may shift investments from safe haven to risky assets. To understand this change in the context of the light-a-lamp event, we examine the behavior of the bond and currency markets in the post-event day. We find a fall (rise) in the bond prices (yields) on the post-event day for the long-term bonds. That is, there is a relatively higher demand for risky assets, which is consistent with our hypothesis. In the case of the currency market, we find exchange rate changes in the expected direction; however, this change is insignificant.

This paper contributes to the pandemic literature in the following ways. The first is the uniqueness of the event during the pandemic time. Even though several other festivals such as Easter and Eid appeared during the lockdown, they mostly cater to a specific group of people. The light-a-lamp event, however, grabbed the attention of the entire country and flooded the related news in the media outlets. Second, we link this event with the stock market. The existing studies during the pandemic largely focus on its adverse effect on stock market (Ali et al. 2020; Haroon and Rizvi 2020a; Mishra et al. 2020; Phan and Narayan 2020; Salisu and Akanni 2020). We focus, however, on a particular event that may alter the behavior of financial asset traders during the pandemic. Because this event happened when most people faced a depressing and challenging time, the psychological relief from this event should be substantial, and thus, its sentiment-led impact on the stock market requires special attention (Edmans et al. 2007; Drakos 2010; Akyildirim et al. 2020). Finally, we provide robust evidence for a stock market boost in the post-event day combined with an adverse effect on the long-term bond yields. In light of limit to arbitrage, we also find immense heterogeneity in the event effect.

The remainder of this paper is organized as follows. "The event and the review of literature" section provides an overview of the event and trace out relevant existing studies. "Data and methodology" section  explains the data and empirical framework. "Results" section presents our estimation findings.  "The event and other financial markets" provides evidence of the impact of the event on bond and currency markets. Finally, "Conclusion" concludes the study.

## The event and the review of literature

### Light-a-lamp event: an overview

To control the spread of COVID-19 across the country, the Indian government declared a lockdown and adopted strict quarantine measures beginning March 24, 2020. The lockdown initially was announced for a period of 21 days and was later extended until the end of May. The social distancing and isolation measures imposed during these days led to spike in the rate of unemployment and people lost their livelihood. These measures also became one of the main reasons for psychological stress among people. In addition, the mental health of people was influenced by depressing news on COVID-19 too. During this disappointing time, the prime minister of India addressed the nation and called for the light-a-lamp event. The event urged the people to switch off all lights in their house and to light a lamp, candle, or mobile flashlight for nine minutes at 9:00 p.m. on April 5, 2020. Although the purpose of the event was to show solidarity and unity in the fight against the pandemic,^2^ it has also provided psychological relief to the people.^3^ Millions of people participated in the event. The following are some of the news headlines from the day after the event.^4^

“Millions of Indians respond to PM's appeal; light candles, diyas, turn on mobile phone torches” (*Economic Times*, April 6, 2020)

“Coronavirus: India holds lights-off vigil as Modi calls for unity” (BBC, April 6, 2020)

“Nine minutes of cheer even as coronavirus cases spiral” (*Live Mint*, April 6, 2020)

“Coronavirus: India lights up to heed Modi's call for unity” (*Al-Jazeera*, April 6, 2020)

### Review of literature

Before the outbreak of COVID-19, pandemic related studies on stock market were largely focused on influenza and SARS. Mctier et al. (2011) examine the reaction of the U.S. stock market to the flu. They argue that the rate of increase in the flu is negatively associated with stock returns. Chen et al. (2007) provide evidence for the stock market plunge in the Taiwan stock market during the SARS outbreak, especially in the hotel industry. In a similar line, Mei-Ping et al. (2018) find that the SARS outbreak weakens the integration of Asian stock markets.

The recent outbreak of COVID-19 gained significant attention in the literature on financial markets. The initial studies exploring the impact of the pandemic have identified a drastic plummet in the stock market (Phan and Narayan 2020; Zhang et al. 2020), and this result is supported by the findings of succeeding studies. Narayan and Phan (2020) find that the lockdown of economic activities globally has been reflected significantly in the stock market. Ali et al. (2020) explores the effect of the pandemic on different financial securities and compares the situation of the Chinese economy with other economies. They find a significant adverse effect of COVID-19 in the financial market, including the commodities market. Haroon and Rizvi (2020a) find the media coverage on the pandemic has significantly increased the volatility in the equity market. The fall in the number of confirmed COVID-19 cases leads to an improvement in market liquidity in the financial market (Haroon and Rizvi 2020b). During this challenging time, Salisu and Sikiru (2020) also find that Islamic stocks serve as a good hedge against the crisis. The peril of the pandemic on Indian markets is not different. A recent study by Mishra et al. (2020) finds that the impact of the current crisis on the Indian stock market is more severe than during the time of demonetization and implementation of the goods and services tax.

Unlike these studies, using a time-varying parameter vector autoregression model, Liu et al. (2020) find a positive reaction of the stock market for the pandemic crisis. In the same fashion, He et al. (2020) find a positive stock market response in the Chinese stock exchange, especially in the manufacturing, information technology, education, and health industries. Furthermore, the impact of the pandemic is observed not only in the stock market but also in other financial markets, such as oil market (e.g., see Qin et al. 2020; Apergis and Apergis 2020; Fu and Shen 2020; Gil-Alana and Monge 2020; Narayan 2020a, b; Huang and Zheng 2020; Devpura and Narayan 2020) and foreign exchange market (Iyke 2020; Narayan 2020a, b). In general, no studies explore the effect of an event that happened during the time of COVID-19 on financial market in response to people’s emotions.

## Data and methodology

### Data

To study the impact of the event on stock market, we use both market index data and firm-level data from CMIE-Prowess for the period from March, 1 to April 30, 2020. The study period includes 39 trading days. Our dataset has both trading-related and accounting measures (as on March 2020). To analyze the event effect on the fixed-income market, we use bond yields of 3-month, 6-month, 1-year, 3-year, 10-year, 15-year, 19-year, 24-year, and 30-year government securities. The bond yields are based on trade that happened in the National Stock Exchange and are collected from investing.com. Finally, we obtain exchange rate data from the Reserve Bank of India database to investigate the effect of event on currency market.

### Methodology

#### The event and stock market reaction

To investigate the sentiment-driven effect of the event on the stock market, similar to Kaplanski and Levy (2010), we estimate the following model:

1 $$r_{t + k} = \alpha + \beta Event_{t} + Controls + \varepsilon_{t + k} ,$$

where *r*_*t*_ is the daily returns at time *t*, calculated as log change of Nifty 50 index. Following Da et al. (2015), *k* takes a value from 1 to 5. *Event* is a dummy variable that takes a value equal to 1 for the post-event trading day (April 7, 2020); zero otherwise. Our control variables include past returns; day-of-the-week and month fixed effects; trading-related variables such as price-to-earnings ratio, price-to-book value ratio, and volume; and trend.^5^ Our main variable of interest is *Event*. If the event under consideration positively influences people’s mood during the difficult time of the lockdown, we expect a positive and significant value for $\beta$. On the contrary, if the event is not sufficient to boost investor sentiment in the market, we expect an insignificant reaction from the stock market.

#### Limit to arbitrage and heterogenous effect

Our next set of analyses investigates the heterogeneous effect of the event on different stocks. For that, we use firm-level data and estimate the following model:

2 $$r_{i,t + k} = \alpha + \beta Event_{t} + \gamma X_{i,t} + \delta Event_{t} *X_{i,t} + Controls + \varepsilon_{i,t + k} ,\quad k = 1 \;{\text{to}}\;5,$$

where *r*_*it*_ is the daily returns of firm *i* at time *t*; and *X* is the set of main firm-level explanatory variables. The set *X* includes beta (Beta), downside risk (DS_Beta), volatility (Volatility), size (Size), and financial distress measures.^6^ The main variable of interest is the interaction between *Event* and *X*. Beta is a risk factor from the capital asset pricing model (Sharpe 1964; Lintner 1965). Following Bawa and Lindenberg (1977), we use downside beta as a proxy for downside risk, measured as follows: $DS\_Beta_{i} = \frac{cov(ri,rm|rm < average\_rm)}{{var(rm|rm < average\_rm)}},$ where *DS_Beta* is the downside risk; *r*_*i*_ and *r*_*m*_ are individual stock and market returns respectively; and *average_r*_*m*_ is the average market return. This measure is constructed using the data in our sample.

The volatility of the return is calculated by Garman and Klass (1980) method, as follows:

$$\begin{aligned}Volatility_{i,t} &= 0.511\ln \left( {\frac{{h_{i,t} }}{{l_{i,t} }}} \right) - 0.019 \left[ {\ln \left( {\frac{{c_{i,t} }}{{o_{i,t} }}} \right)\ln \left( {\frac{{h_{i,t} l_{i,t} }}{{o_{i,t}^{2} }}} \right) - 2\ln \left( {\frac{{h_{i,t} }}{{o_{i,t} }}} \right)ln \left( {\frac{{l_{i,t} }}{{o_{i,t} }}} \right)} \right]\\&\quad - 0.383 \left[ {\ln \left( {\frac{{c_{i,t} }}{{o_{i,t} }}} \right)} \right]^{2} ,\end{aligned}$$

where *h*, *l*, *o*, and *c* are the highest, lowest, opening, and closing prices, respectively. Size of the firm is measured as log of total assets. We use several proxies to account for financial distress of a firm, including cashflow, dividend, Kaplan and Zingales index, and Whited and Wu index (Fazzari et al. 1988; Kaplan and Zingales 1997; Whited and Wu 2006).

$$\begin{aligned} KZI_{i,t} & = { } - 1.001909*Cashflow_{i,2019} + 3.139193*Debt_{i,2019} - 39.36780*Dividend_{i,2019} \\ & \quad - \,1.314759*Cash_{i,2019} + 0.2826389*Tobin^{\prime}s Q_{i,2019} \\ \end{aligned}$$

$$\begin{aligned} WWI_{i,t} & = { } - 0.091*Cashflow_{i,2019} + 0.021*Debt_{i,2019} - 0.062*Dividend_{i,2019} \\ & \quad - \,0.044*Total Assets_{i,2019} - 0.035*SG_{i,2019} + 0.102*ISG_{i,2019} , \\ \end{aligned}$$

where *KZI* is the Kaplan and Zingales index, and *WWI* is the Whited and Wu index. Cashflow is the cashflow measured by the ratio of the sum of profit after tax, depreciation, and amortization to total assets. *Debt* is measured by total debts to total assets. *Dividend* is a dummy variable that takes a value equal to 1 if the firm pays the dividend; zero otherwise. *Cash* is measured as the ratio of total cash in hand to the total assets. Tobin’s *Q* is measured by the ratio of market capitalization to the total assets. *Total Assets* (a proxy for firm size) is measured by the log of total assets. *SG* is the annual sales growth, and *ISG* denotes industry sales growth. To calculate *ISG*, we divide firms into different industry groups based on the 2-digit National Industrial Classification (NIC).

Our analysis includes several trade-related variables and firm characteristics as additional control variables. The trading-related controls include past returns, price-to-earnings ratio (*P*/*E*), price-to-book value ratio (*P*/*B*), log of turnover (*Turnover*), and log of market capitalization (*MarketCap*). We employ the price-to-earnings ratio after it is winsorized at the 1% level on both sides. The firm-specific control variables include size measured by log of total assets (*Size*), age of the firm is measured by the log of number of years since incorporated (*Age*), return on asset is measured by the ratio of profit after tax to total assets (*ROA*), and debt of the firm is measured by ratio of total debt to total asset (*Debt*).

#### The event and other financial markets

To study the impact of event on bond market, we run a regression of the percentage change in the yields on its past value, month dummies, day-of-the-week dummies, and *Event*. Furthermore, to examine the impact of the light-a-lamp event on the currency market, we run a regression of percentage change in the exchange rate (the value of the Indian rupees against U.S. dollars) on its past value, month dummies, day-of-the-week dummies, and *Event*.

## Results

### The event and stock market reaction

We first examine the impact of the light-a-lamp event on the stock market. We analyze this in light of investor sentiments and misattribution bias. If the event influences people’s mood positively, it affects the risk-taking behavior of market participants. As a result, investors demand more assets, and, in the presence of limits to arbitrage, this pushes the asset prices forward. To examine this impact, we estimate Eq. 1.

Table 1 reports the estimation result of Eq. 1. In Panel A, Column 1 reports the most parsimonious specification results of our model. We find that the coefficient of *Event* is positive and statistically significant at the 1% level. On an immediate day after the event, the market has gains above 9% returns compared with other days, which is close to hitting the 10% circuit breaker. This result is consistent with what we observe in Fig. 1. Column 2 of Table 1 reports the estimation result with past returns as a control variable. The inclusion of past returns helps to offset the problem of serial correlation that may arise because of a weak tendency of price movements (Schwert 1990a, b; Fisher 1966; Kaplanski and Levy 2010). Furthermore, to offset “weekend effect” or “Monday effect” (French 1980; Cho et al. 2007), we include dummy variables for the day of the week as an additional control measure. In the same way, we include month dummies to alleviate the month-fixed effect. In Column 3, we examine the impact of the event on the stock market after controlling for the day-of-the-week and month effects. The estimation results imply that the inclusion of control variables does not change our main findings. To check the consistency of our result further, we include trade-related control variables. Since the daily return is also a function of liquidity, we plug market volume (*Volume*) to our model. Apart from that, we also include P/E and P/B (Fama and French 2006). Column 4 shows that our main finding is unaffected by the inclusion of trade-related variables. As a final specification, we control for market trends. The trend line in Fig. 1 indicates an upward stock market movement around the event day. To confirm that our result is not merely the result of an upward trend in the stock market, we include a time variable in our model. The result, given in Column 5 of Table 1, suggests that, consistent with the reported results, a positive correlation exists between *Event* and market returns.^7^ These findings align with the existing studies that show investors’ good mood has a positive impact on the stock market (Kamstra et al. 2003; Chang et al. 2008). In other words, investors become less risk averse and bid risky assets at the time of positive emotions (Johnson and Tversky 1983; Hirshleifer 2001; Baker and Wurgler 2007; DellaVigna 2009).

**Table 1:** Light a lamp and stock market returns

Variables	(1)	(2)	(3)	(4)	(5)
	Returns_t+1_	Returns_t+1_	Returns_t+1_	Returns_t+1_	Returns_t+1_
Panel A: Market reaction to the event					
Event	9.317***	8.930***	7.403***	8.336***	9.153***
	(0.648)	(0.589)	(1.528)	(1.683)	(2.207)
Past returns		− 0.180	− 0.168	− 0.260*	− 0.265*
		(0.157)	(0.169)	(0.150)	(0.153)
P/E				− 14.96***	− 13.09**
				(4.010)	(4.849)
P/B				129.3***	114.6***
				(33.51)	(39.82)
Volume				2.242	1.394
				(3.034)	(3.589)
Time					0.0852
					(0.114)
Constant	− 0.557	− 0.541	− 9.309*	− 50.87	− 38.19
	(0.648)	(0.684)	(5.340)	(40.46)	(48.38)
Day of the week dummies	No	Yes	Yes	Yes	Yes
Month dummies	No	Yes	Yes	Yes	Yes
Observations	39	39	39	39	39
R-squared	0.122	0.154	0.338	0.569	0.574

Variables	Returns_t+2_	Returns_t+3_	Returns_t+4_	Returns_t+5_
Panel B: Reversal				
Event	− 2.583	4.506	− 7.790**	3.200
	(2.606)	(2.853)	(3.723)	(3.264)
Controls	Yes	Yes	Yes	Yes
Observations	38	37	36	35
R-squared	0.279	0.460	0.462	0.392

This table shows the estimation result of $r_{t + k} = \alpha + \beta Event_{t} + Controls + \varepsilon_{t + k}$ to explore the impact of *light a lamp* event on the stock market. Panel A uses returns at time *t* + 1 (*k* = 1) and Panel B uses returns at time *t* + 2 to *t* + 5 (*k* = 2 to 5). *Event* is a dummy variable that take value 1 for the post-event trading day; zero otherwise. *Controls* includes past returns, day of the week and month fixed effects; trading related variables such as price-to-earnings ratio (*P*/*E*), price-to-book value ratio (*P*/*B*) and volume and trend (*Time*). Robust standard errors in parentheses ***p < 0.01, **p < 0.05, *p < 0.1.

We observed a stock market boost on the day after the event. This effect may be due to the positive sentiments and changes in the risk-taking behavior of market participants. The sentiment-driven price movements, however, do not persist for a long time, and they revert over the period (Tetlock 2007; Garcia 2013). Because the light-a-lamp event does not hold economic importance, the chance of price reversal is higher. To test this, we explore the behavior of market returns until the fifth day after the event. Panel B of Table 1 reports the estimation result, and we observe a reversal on the fourth day, which is consistent with sentiment-induced temporary mispricing (Da et al. 2015). Specifically, we observe a fall of around 8% returns in Nifty 50 at *k* = 4. Because the sentiment-driven hike in the stock market is around 9% at *k* = 1, we do not observe a complete reversal. The coefficient of *Event* is insignificant on the other days. We find two possible explanations for the return reversal in the following days. The first one is overreaction (Lee et al. 1991; Kaplanski and Levy 2010); that is, investors become overly enthusiastic after the event and start bidding on assets beyond their economic rationale, and they rectify their mistakes in the following days. Another explanation stems from cognitive ease and cognitive strain theories (Kahneman 2011). That is, investors may make mistakes in a relaxed environment after the event and correct these mistakes in the following trading days.

### Limit to arbitrage and heterogeneous effect

Previous findings show that the sentiment effects are not uniform across the stocks (Baker and Wurgler 2006, 2007). One reason for the differential effect is the limit to arbitrage (Pontiff 1996; Shleifer and Vishny 1997). The arbitrageurs try to take advantage of sentiment-led mispricing. This advantage is limited, however, to a certain set of assets. Therefore, we estimate Eq. 2 and extend our analysis to explore the impact of the light-a-lamp event on stocks that have different characteristics.

#### Beta

We next investigate the heterogeneous impact of the event on stocks with different beta. Existing studies present evidence that high-beta stocks are more sensitive to investor sentiment (Baker et al. 2011). Therefore, we believe that the sentiment-driven effect of the light-a-lamp event may have a larger impact on high-beta stocks. Table 2 reports the estimation results. Consistent with Baker et al. (2011), we find evidence for a higher sentiment effect on high-beta stocks the day after the event. The interaction between the event measure beta is positive and statistically significant at conventional levels. More specifically, an increase of one standard deviation of beta is associated with 1.52% of a higher return on the immediate day of the event. When we extend *k*, we find evidence for the persistence of this sentiment effect. This result aligns with the existing studies that high-beta stocks are off-limits for arbitrageurs.

**Table 2:** Light a lamp, beta and returns

Variables	(1)	(2)	(3)	(4)	(5)
	Returns_t+1_	Returns_t+2_	Returns_t+3_	Returns_t+4_	Returns_t+5_
Event*Beta	2.610***	1.432***	2.569***	0.0378	0.452*
	(0.282)	(0.269)	(0.263)	(0.290)	(0.260)
Past returns	− 0.0205***	0.0735***	0.0697***	0.0357***	0.0899***
	(0.00753)	(0.00611)	(0.00589)	(0.00548)	(0.00601)
Event	− 0.250	− 0.934***	− 0.939***	− 2.365***	1.803***
	(0.341)	(0.327)	(0.324)	(0.359)	(0.333)
Beta	0.117**	− 0.0295	− 0.0602	0.0252	− 0.00654
	(0.0503)	(0.0461)	(0.0464)	(0.0487)	(0.0474)
Age	0.0473	0.0645*	0.0624*	0.0497	0.0463
	(0.0358)	(0.0334)	(0.0346)	(0.0364)	(0.0360)
Size	− 0.119***	0.0693***	0.0871***	0.104***	0.110***
	(0.0243)	(0.0257)	(0.0269)	(0.0294)	(0.0302)
P/E	− 7.84e−05	− 0.000219	− 0.000471	− 0.000556	− 0.000603*
	(0.000348)	(0.000317)	(0.000318)	(0.000357)	(0.000358)
P/B	0.00878*	0.0132***	0.0183***	0.0198***	0.0178***
	(0.00464)	(0.00448)	(0.00522)	(0.00571)	(0.00550)
Turnover	0.132***	0.0252	− 0.0338	0.00207	0.00394
	(0.0295)	(0.0265)	(0.0273)	(0.0277)	(0.0278)
MarketCap	0.0224	− 0.108***	− 0.110***	− 0.149***	− 0.153***
	(0.0215)	(0.0226)	(0.0238)	(0.0262)	(0.0268)
ROA	0.223	1.419***	1.470***	1.716***	1.605***
	(0.353)	(0.353)	(0.371)	(0.393)	(0.385)
Debt	− 0.0803	− 0.230*	− 0.243*	− 0.274*	− 0.305*
	(0.102)	(0.126)	(0.136)	(0.153)	(0.161)
Constant	− 2.594***	− 0.990***	− 2.057***	− 0.234	− 0.516***
	(0.180)	(0.166)	(0.170)	(0.180)	(0.178)
Observations	49,234	46,322	44,932	43,597	42,272

This table shows the estimation result of $r_{i,t + k} = \alpha + \beta Event_{t} + \gamma Beta_{i,t} + \delta Event_{t} *Beta_{i,t} + Controls + \varepsilon_{i,t + k}$ to explore the differential effect of *light a lamp* event on the stocks with different beta. *Event* is a dummy variable that takes value 1 for the post-event trading day; zero otherwise. *Beta* is the CAPM risk factor. *Controls* include past returns, price-to-earnings ratio (*P*/*E*), price-to-book value ratio (*P*/*B*), log of turnover (*Turnover*) and log of market capitalization (*MarketCap*), log of total assets (*Size*), age of the firm measured by the log of number of years since incorporated (*Age*), return on asset measured by the ratio of profit after tax to total assets *ROA*, and debt of the firm measured by the ratio of total debt to total asset (*Debt*). Robust standard errors in parentheses ***p < 0.01, **p < 0.05, *p < 0.1.

Turning toward the other variables in Column 1 of Table 2, the coefficient of *Event* and *Beta* is positive and statistically significant. This result indicates that higher beta is associated with higher returns irrespective of whether it is an event or a nonevent day. This result is consistent with the classic CAPM model (Sharpe 1964; Lintner 1965). The positive coefficient of *Event* indicates that the return boost is not only subject to high-beta stock but also for low-beta stocks; however, the effect is more pronounced in the high-beta firms. The coefficient of size indicates that there is a size premium for relatively small firms (Banz 1981). The older firms receive relatively lower returns compared with larger firms; however, this effect is not statistically significant.

#### Downside risk

Even though CAPM beta accounts for overall market risk, there is considerable criticism in capturing downside risk (Ang et al. 2006). As a result, this section examines the investor sentiment effect on various downside risk stocks. In particular, we investigate whether the returns are different for stocks with different levels of downside risk in the post-event days. Table 3 reports the estimation result of Eq. 2 using downside risk. The main variable of interest in this analysis is the interaction between Event and DS_Beta. The coefficient of the interaction term suggests that the sentiment-led return is more pronounced in the stocks with a relatively higher downside risk. A stock with one standard deviation higher downward risk is associated with 2.4% higher returns at *k* = 1.

**Table 3:** Light a lamp, downside risk and returns

Variables	(1)	(2)	(3)	(4)	(5)
	Returns_t+1_	Returns_t+2_	Returns_t+3_	Returns_t+4_	Returns_t+5_
Event*DS_Beta	0.0322***	0.0219***	0.0237***	− 0.000981	0.0112***
	(0.00655)	(0.00350)	(0.00370)	(0.00382)	(0.00348)
Past returns	− 0.0120	0.0755***	0.0714***	0.0369***	0.0899***
	(0.00744)	(0.00602)	(0.00573)	(0.00533)	(0.00584)
Event	0.490	− 0.795***	0.352	− 2.338***	1.555***
	(0.434)	(0.262)	(0.295)	(0.291)	(0.275)
DS_Beta	− 0.00344***	− 0.00262***	− 0.00279***	− 0.00187***	− 0.00240***
	(0.000691)	(0.000671)	(0.000692)	(0.000714)	(0.000698)
Age	0.0943***	0.0812***	0.0780***	0.0802**	0.0840***
	(0.0291)	(0.0288)	(0.0298)	(0.0313)	(0.0303)
Size	− 0.0921***	0.0719***	0.0902***	0.117***	0.124***
	(0.0207)	(0.0224)	(0.0238)	(0.0266)	(0.0270)
P/E	− 0.000183	− 0.000402	− 0.000601*	− 0.000710**	− 0.000809**
	(0.000301)	(0.000302)	(0.000307)	(0.000343)	(0.000349)
P/B	0.00145	0.0157***	0.0182***	0.0219***	0.0220***
	(0.00272)	(0.00298)	(0.00326)	(0.00360)	(0.00354)
Turnover	0.103***	0.0267	− 0.0380	− 0.00601	− 0.00360
	(0.0251)	(0.0232)	(0.0241)	(0.0246)	(0.0248)
MarketCap	0.0374*	− 0.102***	− 0.0992***	− 0.144***	− 0.152***
	(0.0198)	(0.0213)	(0.0226)	(0.0252)	(0.0254)
ROA	0.578**	1.415***	1.511***	1.735***	1.595***
	(0.290)	(0.312)	(0.330)	(0.361)	(0.346)
Debt	− 0.0338	− 0.209*	− 0.224*	− 0.244*	− 0.277*
	(0.0974)	(0.113)	(0.123)	(0.139)	(0.147)
Constant	− 2.755***	− 0.959***	− 2.129***	− 0.331**	− 0.614***
	(0.159)	(0.156)	(0.158)	(0.167)	(0.165)
Observations	53,099	49,769	48,304	46,875	45,469

This table shows the estimation result of $r_{i,t + k} = \alpha + \beta Event_{t} + \gamma {\text{DS}}\_{\text{Beta}}_{i,t} + \delta Event_{t} *{\text{DS}}\_{\text{Beta}}_{i,t} + Controls + \varepsilon_{i,t + k}$ to explore the differential effect of *light a lamp* event on the stocks with different downside risk. *Event* is a dummy variable that takes value 1 for the post-event trading day; zero otherwise. *DS_Beta* is downside beta-proxy for downside risk. *Controls* include past returns, price-to-earnings ratio (*P*/*E*), price-to-book value ratio (*P*/*B*), log of turnover (*Turnover*) and log of market capitalization (*MarketCap*), log of total assets (*Size*), age of the firm measured by the log of number of years since incorporated (*Age*), return on asset measured by the ratio of profit after tax to total assets *ROA*, and debt of the firm measured by the ratio of total debt to total asset (*Debt*). Robust standard errors in parentheses ***p < 0.01, **p < 0.05, *p < 0.1.

#### Volatility

Another proxy for the limit to arbitrage is the return volatility (Wurgler and Zhuravskaya 2002). Da et al. (2015) find that the sentimental effect is higher for high-volatility stocks. Based on that finding, we investigate the differential impact of the light-a-lamp sentiments on returns of low- and high-volatility stocks. Table 4 reports the estimation result of Eq. 2 with volatility. This result shows that the event has a higher impact on relatively high-volatility stock on the day immediately after the event. At *k* = 1, stocks with one-standard-deviation-higher volatility gain 1.45% more return than their counterparts. This result is consistent with the findings of Da et al. (2015); that is, the sentimental effect is relatively higher on high-volatility stocks. Similar to other risk measures, we observe a price reversal on the fourth trading day after the event.

**Table 4:** Light a lamp, volatility and returns

Variables	(1)	(2)	(3)	(4)	(5)
	Returns_t+1_	Returns_t+2_	Returns_t+3_	Returns_t+4_	Returns_t+5_
Event*Volatility	0.485***	0.0965	0.140**	− 0.125**	− 0.0566
	(0.0824)	(0.0675)	(0.0641)	(0.0558)	(0.0517)
Event	1.140***	0.377	1.572***	− 1.892***	2.522***
	(0.252)	(0.234)	(0.243)	(0.237)	(0.219)
Volatility	− 0.0477***	0.0493***	0.0188*	0.00666	0.0187*
	(0.0109)	(0.00996)	(0.0101)	(0.0101)	(0.00962)
Past returns	− 0.0215***	0.0751***	0.0708***	0.0358***	0.0906***
	(0.00750)	(0.00610)	(0.00589)	(0.00545)	(0.00600)
Age	0.0465	0.0594*	0.0583*	0.0496	0.0448
	(0.0358)	(0.0332)	(0.0343)	(0.0364)	(0.0359)
Size	− 0.122***	0.0749***	0.0881***	0.105***	0.112***
	(0.0241)	(0.0268)	(0.0272)	(0.0295)	(0.0306)
Beta	0.210***	− 0.0268	− 0.00912	0.0235	− 0.00528
	(0.0502)	(0.0462)	(0.0462)	(0.0483)	(0.0468)
P/E	− 0.000154	− 0.000100	− 0.000417	− 0.000550	− 0.000563
	(0.000347)	(0.000322)	(0.000318)	(0.000357)	(0.000358)
P/B	0.00732	0.0143***	0.0185***	0.0200***	0.0182***
	(0.00456)	(0.00476)	(0.00532)	(0.00576)	(0.00561)
Turnover	0.139***	0.00861	− 0.0405	0.00163	− 0.00104
	(0.0287)	(0.0267)	(0.0271)	(0.0275)	(0.0276)
MarketCap	0.0213	− 0.102***	− 0.106***	− 0.149***	− 0.151***
	(0.0214)	(0.0234)	(0.0241)	(0.0263)	(0.0270)
ROA	0.327	1.341***	1.442***	1.708***	1.576***
	(0.349)	(0.362)	(0.373)	(0.394)	(0.386)
Age	− 0.0708	− 0.239*	− 0.243*	− 0.275*	− 0.307*
	(0.102)	(0.138)	(0.141)	(0.153)	(0.165)
Constant	− 2.458***	− 1.292***	− 2.228***	− 0.266	− 0.627***
	(0.187)	(0.173)	(0.178)	(0.188)	(0.183)
Observations	49,234	46,322	44,932	43,597	42,272

This table shows the estimation result of $r_{i,t + k} = \alpha + \beta Event_{t} + \gamma {\text{Volatility}}_{i,t} + \delta Event_{t} *{\text{Volatility}}_{i,t} + Controls + \varepsilon_{i,t + k}$ to explore the differential effect of *light a lamp* event on the stocks with different volatility levels. *Event* is a dummy variable that takes value 1 for the post-event trading day; zero otherwise. Volatility is a measure of stock volatility. *Controls* include past returns, price-to-earnings ratio (*P*/*E*), price-to-book value ratio (*P*/*B*), log of turnover (*Turnover*) and log of market capitalization (*MarketCap*), log of total assets (*Size*), age of the firm measured by the log of number of years since incorporated (*Age*), return on asset measured by the ratio of profit after tax to total assets *ROA*, and debt of the firm measured by the ratio of total debt to total asset (*Debt*). Robust standard errors in parentheses ***p < 0.01, **p < 0.05, *p < 0.1

#### Size and financial distress

Investor sentiment is higher in smaller firms for which the opportunity for arbitrage is limited (Baker and Wurgler 2006; Wurgler and Zhuravskaya 2002). Kaplanski and Levy (2010) show that evidence for sentiment is more pronounced in small firms. As a result, we extend our study to explore the heterogeneous effects of sentiments on small and large firms. In other words, we study whether the impact of the light-a-lamp event is different across firms of different sizes. Table 5 reports the estimation result. Unlike the existing studies, we find that larger firms reap greater returns on the post-event days than smaller firms. For a standard deviation increase in the size measure, we find a 1.23% higher return on the immediate post-event day.^8^

**Table 5:** Light a lamp, size and returns

Variables	(1)	(2)	(3)	(4)	(5)
	Returns_t+1_	Returns_t+2_	Returns_t+3_	Returns_t+4_	Returns_t+5_
Event*Size	0.463***	0.0575	0.239***	− 0.128**	0.0332
	(0.0712)	(0.0621)	(0.0660)	(0.0631)	(0.0574)
Event	− 1.308**	0.238	− 0.0481	− 1.183**	2.041***
	(0.648)	(0.583)	(0.636)	(0.600)	(0.550)
Size	− 0.129***	0.0674***	0.0793***	0.108***	0.109***
	(0.0243)	(0.0256)	(0.0269)	(0.0296)	(0.0302)
Past returns	− 0.0196***	0.0739***	0.0704***	0.0356***	0.0900***
	(0.00753)	(0.00611)	(0.00589)	(0.00548)	(0.00601)
Beta	− 7.51e−05	− 0.000215	− 0.000468	− 0.000554	− 0.000601*
	(0.000347)	(0.000317)	(0.000318)	(0.000357)	(0.000358)
P/E	0.00867*	0.0131***	0.0181***	0.0198***	0.0177***
	(0.00461)	(0.00446)	(0.00517)	(0.00571)	(0.00549)
P/B	0.131***	0.0255	− 0.0324	0.00185	0.00415
	(0.0293)	(0.0264)	(0.0270)	(0.0277)	(0.0277)
Turnover	0.0217	− 0.108***	− 0.109***	− 0.149***	− 0.153***
	(0.0213)	(0.0225)	(0.0237)	(0.0262)	(0.0268)
MarketCap	0.250	1.426***	1.481***	1.717***	1.608***
	(0.352)	(0.352)	(0.370)	(0.394)	(0.384)
ROA	0.0458	0.0634*	0.0604*	0.0494	0.0459
	(0.0356)	(0.0333)	(0.0344)	(0.0364)	(0.0360)
Age	− 0.0786	− 0.228*	− 0.239*	− 0.274*	− 0.304*
	(0.102)	(0.126)	(0.136)	(0.153)	(0.161)
Debt	− 2.561***	− 1.015***	− 2.067***	− 0.268	− 0.520***
	(0.179)	(0.165)	(0.168)	(0.179)	(0.176)
Constant	− 7.51e−05	− 0.000215	− 0.000468	− 0.000554	− 0.000601*
	(0.000347)	(0.000317)	(0.000318)	(0.000357)	(0.000358)
Observations	49,234	46,322	44,932	43,597	42,272

This table shows the estimation result of $r_{i,t + k} = \alpha + \beta Event_{t} + \gamma {\text{Size}}_{i,t} + \delta Event_{t} *{\text{Size}}_{i,t} + Controls + \varepsilon_{i,t + k}$ to explore the differential effect of *light a lamp* event on the stocks with different size. *Event* is a dummy variable that takes value 1 for the post-event trading day; zero otherwise. Size is log of total assets. *Controls* include past returns, price-to-earnings ratio (*P*/*E*), price-to-book value ratio (*P*/*B*), log of turnover (*Turnover*) and log of market capitalization (*MarketCap*), age of the firm measured by the log of number of years since incorporated (*Age*), return on asset measured by the ratio of profit after tax to total assets *ROA*, and debt of the firm measured by the ratio of total debt to total asset (*Debt*). Robust standard errors in parentheses ***p < 0.01, **p < 0.05, *p < 0.1.

This result may be due to the fact that market participants are looking for sound companies to invest in, particularly given that size is a proxy for financial distress (Hadlock and Pierce 2010). To check whether investors are avoiding distressed firms, we use alternative measures of financial distress (cashflow, dividend, KZI, and WWI) in Eq. 2 (Fazzari et al. 1988; Kaplan and Zingales 1997; Whited and Wu 2006).

Table 6 reports the estimation results using various financial distress measures. Panel A uses cashflow, which is a conventional measure of financial distress. A smaller cashflow indicates higher distress. At *k* = 1, the coefficient of interaction between *Event* and cashflow is positive and statistically significant. One can infer that the demand for the least distressed firms increases in the post-event day more than demand for the distressed firms. This finding remains consistent with alternative proxies for distress. In Panels B, C, and D, we interact *Event* with dividend, KZI, and WWI, respectively.^9^ The coefficient of the interaction is positive in Panel B and negative in Panels C and D, which suggests that the returns from the least distressed firms are relatively higher than the returns from the distressed firms.

**Table 6:** Light a lamp, financial distress and returns

Variables	(1)	(2)	(3)	(4)	(5)
	Returns_t+1_	Returns_t+2_	Returns_t+3_	Returns_t+4_	Returns_t+5_
Panel A: Cashflow					
Event*Cashflow	10.85***	4.152**	− 0.580	− 0.212	2.720
	(2.255)	(1.818)	(2.036)	(2.237)	(1.813)
Controls	Yes	Yes	Yes	Yes	Yes
Observations	48,489	45,692	44,327	43,018	41,717

Variables	(1)	(2)	(3)	(4)	(5)
	Returns_t+1_	Returns_t+2_	Returns_t+3_	Returns_t+4_	Returns_t+5_
Panel B: Dividend					
Event*Dividend	1.224***	0.205	0.967***	− 0.112	0.460
	(0.312)	(0.296)	(0.310)	(0.317)	(0.292)
Controls	Yes	Yes	Yes	Yes	Yes
Observations	49,234	46,322	44,932	43,597	42,272

Variables	(1)	(2)	(3)	(4)	(5)
	Returns_t+1_	Returns_t+2_	Returns_t+3_	Returns_t+4_	Returns_t+5_
Panel C: Kaplan and Zingales index					
Event*KZI	− 0.0312***	-0.00567	− 0.0232***	0.000681	− 0.0112
	(0.00858)	(0.00795)	(0.00856)	(0.00883)	(0.00823)
Controls	Yes	Yes	Yes	Yes	Yes
Observations	43,062	40,630	39,426	38,274	37,111

Variables	(1)	(2)	(3)	(4)	(5)
	Returns_t+1_	Returns_t+2_	Returns_t+3_	Returns_t+4_	Returns_t+5_
Panel D: Whited and Wu index					
Event*WWI	− 5.809**	− 0.350	− 4.184***	2.800**	− 1.444
	(2.405)	(1.689)	(1.229)	(1.218)	(1.072)
Controls	Yes	Yes	Yes	Yes	Yes
Observations	45,768	43,181	41,892	40,668	39,451

This table shows the estimation result of $r_{i,t + k} = \alpha + \beta Event_{t} + \gamma {\text{Financial Distress}}_{i,t} + \delta Event_{t} *{\text{Financial Distress}}_{i,t} + Controls + \varepsilon_{i,t + k}$ to explore the differential effect of *light a lamp* event on the stocks with different financial distress levels. *Event* is a dummy variable that takes value 1 for the post-event trading day; zero otherwise. Panel A, B, C and D use cashflow, dividend, Kaplan and Zingales (1997) index and Whited and Wu (2006) index respectively as distress measures. *Controls* include past returns, price-to-earnings ratio (*P/E)*, price-to-book value ratio (*P/B)*, log of turnover (*Turnover)* and log of market capitalization *(MarketCap)*, log of total assets (*Size)*, age of the firm measured by the log of number of years since incorporated *(Age)*, return on asset measured by the ratio of profit after tax to total assets *ROA*, and debt of the firm measured by the ratio of total debt to total asset (*Debt)*. We included only trading related variables as controls while employing KZI and WWI since other measures are used to construct the distress indices Robust standard errors in parentheses *** p < 0.01, ** p < 0.05, * p < 0.1.

## The event and other financial markets

This analysis presents robust evidence that the light-a-lamp event significantly changed people’s moods, which is reflected in the stock market through investor sentiment. We examine whether the overly enthusiastic market participants move their investments from safe assets to risky assets. For that, we extend our analysis to other financial markets.

First, we test the impact of the event on bond yields. If investors shift their investment for bonds to stocks, then the demand for bonds reduces, and we expect to observe a price (yield) fall (rise) on the post-event day. In this analysis, we use bond yields of 3-month, 6-month, 1-year, 3-year, 10-year, 15-year, 19-year, 24-year, and 30-year government securities. Panel A of Table 7 provides the regression result of the percentage change in the yields on its past value, month dummies, day-of-the-week dummies, and *Event*. The result indicates that there is a significant fall in the bond prices (or increased bond yields) of long-term bonds. The coefficient of *Event* is positive and statistically significant for the bonds with 19- to 30-year maturity periods. This finding aligns with our hypothesis that market participants are more willing to take risks when they are in a good mood. On the contrary, the coefficient of our main variable is statistically insignificant for the other bonds.

**Table 7:** Light a lamp, bond yields and exchange rate

Variables	Event		Controls	Observations	R-squared
Panel A: Bond					
3-Month	− 5.773	(3.800)	Yes	36	0.208
6-Month	− 0.972	(2.913)	Yes	36	0.272
1-Year	− 1.006	(3.059)	Yes	36	0.378
3-Year	1.179	(1.968)	Yes	36	0.237
5-Year	2.908	(2.845)	Yes	36	0.100
10-Year	2.813	(1.696)	Yes	36	0.133
15-Year	1.561	(1.229)	Yes	36	0.087
19-Year	2.411*	(1.216)	Yes	36	0.236
24-Year	2.723**	(1.090)	Yes	36	0.216
30-Year	2.709***	(0.975)	Yes	36	0.288
Panel B: Exchange rate					
USD/INR	0.0100	(0.038)	Yes	36	0.106

Panel A provides the regression result of the percentage change in the yields on its past value, month dummies, day of the week dummies and *Event*. Yields of 3-month, 6-month, 1-year, 3-year, 10-year, 15-year, 19-year, 24-year and 30-year government securities are used. Panel B provides a regression result of the percentage change in the exchange rate (USD/INR) on its past value, month dummies, day of the week dummies and *Event*. *Event* is a dummy variable that takes value 1 for the post-event trading day; zero otherwise. Robust standard errors in parentheses ***p < 0.01, **p < 0.05, *p < 0.1

Second, we examine the impact of the light-a-lamp event on the currency market. We test whether there is an event effect in the value of the Indian rupees against U.S. dollars. If investors withdraw money from the currency market to invest in the equity market, we expect to find a decrease in the value of Indian currency. To test that, we run a regression of percentage change in the exchange rate on its past value, month dummies, day-of-the-week dummies, and *Event*. Panel B of Table 7 shows a rupee depreciation on the immediate day of the event, which is in line with the expected results; however, the coefficient is not statistically significant.

## Conclusion

This study investigates the impact of the light-a-lamp event in India that was held during the COVID-19 lockdown. The event urged people to switch off the lights in their homes and light a lamp for nine minutes on April 5 at 9:00 p.m. This study links this event with the stock market through investor sentiment and misattribution bias. We observe sentiment-driven stock market movement in the post-event day. There was approximately a 9% higher return on the immediate day of the event compared to other days. Since investor sentiment causes this effect, we see a reversal on the fourth day following the event, which is consistent with sentiment-induced temporary mispricing. We extend this study to identify the heterogeneous effect of the event. Consistent with the limit-to-arbitrage literature, we find the effect is more prominent on stocks with high beta, downside risk, and return volatility. In addition, we find that the effect is more pronounced on the least distressed firms. Furthermore, we investigate the impact of the event on bond and currency markets. We find a fall in the long-term bond prices on the post-event day. In the case of U.S. dollar–Indian rupee exchange rate, we find an insignificant effect of the event. To further analyze the behavior of financial markets, this study leaves room to extend future work to use more advanced methods, such as machine learning and internet search frequency (Wen et al. 2019; Kou et al. 2019).

## Appendix

See Table 8.

**Table 8:** Descriptive statistics

Variable	Mean	Std. Dev	Min	Max
Index data				
Returns	− 0.237	4.228	− 12.980	8.760
Event	0.026	0.160	0.000	1.000
P/E	20.916	2.272	17.150	25.730
P/B	2.627	0.263	2.170	3.180
Volume	13.090	0.163	12.672	13.391
Firm-level data				
Returns	− 0.338	5.373	− 72.710	45.070
Event	0.026	0.159	0.000	1.000
Beta	1.172	0.491	− 0.880	2.620
DS_Beta	68.191	38.587	− 134.032	200.184
Volatility	3.825	2.920	0.000	22.171
Size	8.816	2.091	0.095	17.421
Cashflow	0.084	0.067	− 0.314	0.614
Dividend	0.683	0.465	0.000	1.000
WWI	− 0.432	0.114	− 2.897	− 0.087
KZI	− 26.538	18.112	− 39.560	6.436
Debt	0.214	0.192	0.000	2.596
Cash	0.002	0.009	0.000	0.192
Tobin’s Q	1.137	3.897	0.002	144.854
Industry sales growth	0.090	0.082	− 0.471	0.282
Sale growth	0.276	1.840	− 0.968	75.619
Age	3.475	0.522	0.693	5.050
P/E	24.817	54.212	0.750	480.000
P/B	1.800	3.212	0.010	73.790
Turnover	0.861	1.335	0.000	11.625
MarketCap	8.084	2.430	1.348	16.046
ROA	0.061	0.063	− 0.455	0.601
Other financial markets				
3-Month	− 0.925	3.443	− 16.814	6.758
6-Month	− 0.951	2.762	− 10.213	4.569
1-Year	− 0.773	2.945	− 10.663	5.523
3-Year	− 0.487	1.814	− 4.576	2.938
5-Year	− 0.342	2.338	− 6.257	3.488
10-Year	− 0.105	1.386	− 2.670	2.700
15-Year	− 0.093	1.022	− 1.712	2.242
19-Year	− 0.048	1.127	− 2.656	2.412
24-Year	− 0.031	0.973	− 1.744	2.483
30-Year	− 0.070	0.922	− 1.789	2.426
USD/INR	0.108	0.570	− 1.206	1.187

## Footnotes

1 WHO (2020) has advised paying little attention to COVID-19 news to reduce stress and anxiety.

2 The event received considerable criticism in terms of (a) avoiding real issues, (b) being unscientific, and (c) gathering of people during the time of lockdown. The objective of this paper is not to assess whether this was a good or bad event, but rather to evaluate investor sentiment following the light-a-lamp event.

3 The event received international attention in terms of boosting India’s spirit. See https://www.business-standard.com/article/news-ani/pm-modi-boosting-india-s-spirit-to-fight-covid-19-say-experts-120040300784_1.html. Many people participated in the event with devotional songs, mantras, and national anthem. See pictures https://www.theweek.in/news/entertainment/2020/04/06/in-pics-celebrities-light-candles-diyas-participate-modi-initiative.html?utm_source=dlvr.it&utm_medium=facebook.

4 On the day of the event, the hashtag on the event was one of the top Twitter trends. See https://www.livemint.com/news/india/modi-s-covid-19-call-for-sunday-lights-up-twitter-india-with-multiple-trends-11586105414011.html.

5 The descriptive statistics of the variables are provided in Table 8 in “Appendix”.

6 Recent studies (Narayan et al. 2016; Devpura et al. 2018) have suggested combining macroeconomic variables along with firm-level variables in the stock market analysis. Because we are using panel data model along with daily data of 39 trading days, these measures are omitted in the estimation process and are not included in the analysis.

7 Since this event was held at night, there is a possibility for reflecting the sentiment effect in the beginning of the next trading day. To test this possibility, we use returns from the previous day’s closing price and the next day’s opening price. We find a positive coefficient of the event dummy, and it is statistically significant at conventional levels. Results are available upon request.

8 This finding remains robust when we employ market capitalization as a proxy for size, not reported for brevity.

9 We included only trading-related variables as controls while employing KZI and WWI because other measures are used to construct the distress indices.
